# Tooth Extraction Outcomes and Complications in Diabetic and Nondiabetic Individuals: A Systematic Review and Meta‐Analysis to Inform Evidence‐Based Guidelines

**DOI:** 10.1155/ijod/6899990

**Published:** 2026-08-05

**Authors:** Anupam Singh, Srikanth Gadicherla, Kalyana C. Pentapati, Mehul Saha, Premalatha K. Shetty, Aditya Shetty

**Affiliations:** ^1^ Department of Oral and Maxillofacial Surgery, Manipal College of Dental Sciences, Manipal Academy of Higher Education (MAHE), Manipal, Karnataka, India, manipal.edu; ^2^ Department of Public Health Dentistry, Manipal College of Dental Sciences, Manipal Academy of Higher Education (MAHE), Manipal, Karnataka, India, manipal.edu; ^3^ Department of Oral and Maxillofacial Surgery, Manipal College of Dental Sciences Mangalore, Manipal Academy of Higher Education (MAHE), Manipal, Karnataka, India, manipal.edu; ^4^ Department of Conservative Dentistry and Endodontics, Nitte (Deemed to be University), A.B.Shetty Memorial Institute of Dental Sciences (ABSMIDS), Mangalore, Karnataka, India, nitte.edu.in

**Keywords:** diabetes, dry socket, extraction, glycemic control, socket healing, Sustainable Development Goal 3 (Good Health and Well-Being), tooth extraction guidelines

## Abstract

**Background:**

Diabetes mellitus is known to cause long‐term microvascular and macrovascular changes. These changes along with compromised inflammatory response have been known to affect the wound healing. However, the influence of diabetes on the healing process after tooth extraction has not been clearly established. With this background, this systematic review was conducted to compare the healing of extraction sockets and postoperative complications between diabetics and nondiabetics (control).

**Methodology:**

This systematic review was conducted as per the “MOOSE guidelines.” A systematic search was performed in “PubMed,” “Scopus,” “EMBASE,” and “Dentistry and Oral Sciences Source” databases. A comprehensive literature search that was conducted across multiple electronic databases yielded a total of 821 results. After removal of duplicates (*n* = 135) and screening, a total of five publications were included for data extraction.

**Results:**

A total of 500 diabetics and 364 controls were available from five included publications. Adjusted analysis reported that the mean socket size was larger among diabetics than the control group at the end of day 7. There was no significant difference in the distribution of delayed wound healing among diabetics and controls. With regard to complications like dry socket, bony sequestra, and infection, no statistical significance was established. On risk of bias assessment, one study was high quality, one was low quality, and the remaining three were of moderate quality.

**Conclusion:**

There is a lack of scientific evidence to suggest that diabetic patients with well‐controlled glycemic status may have poor healing or increased risk of complications after dental extraction. Overall, there is a need for high‐quality studies and the need for coordinated, multidisciplinary, and transnational efforts among relevant stakeholders to develop clear, evidence‐based guidelines to address this gap and clinical ambiguity. In this context, based on our findings, we have proposed stage‐wise recommendations to streamline the management of diabetic patients during routine dental procedures, ensuring seamless and evidence‐based workflow.

## 1. Introduction

The Global Burden of Disease Study of Diabetes reported that 529 million people are living with diabetes worldwide [1]. Diabetes is a complex metabolic disorder characterized by chronic hyperglycemia resulting from defects in insulin secretion and/or insulin action. Patients with poorly controlled diabetes status can suffer from a range of complications which can be broadly categorized in three different categories—macrovascular, microvascular, and neuropathic [2]. Regardless of the type of diabetes, there is a profound vascular alteration leading to compromised inflammatory response, which includes decreased leukocyte migration, tissue perfusion, and impaired hyperemia, due to which diabetics are always at risk of infections and delayed healing [3, 4].

The alteration in the microvasculature alters the local microcirculation, which is required for adequate nutrition to the tissues as well as removal of waste products [5]. The changes in the microcirculation predispose a diabetic patient to increased chances of infection and/or delay in wound healing following any surgical procedure [6]. The exposure to saliva and other fluids in the oral cavity further compounds the problem. The presence of oral microflora in the saliva makes the tooth extraction site an unsterile environment and increases the chance of postextraction complications like dry socket [7]. Another pathogenesis for increased inflammation is the release of reactive oxygen species (ROS). Increased ROS formation by the polymorphonuclear neutrophils leads to endothelial dysfunction and further tissue damage [8].

Due to the contemporary views, oral health care providers defer diabetics for dental extractions. The influence of diabetes on the healing process after tooth extraction was not clearly established in the published studies [9–16]. Studies in the past evaluated multiple outcomes like delayed healing [10, 12, 15], infection [11, 13, 15], dry sockets [13], bony sequestrum [13, 15], granulation tissue [10], epithelization [10], and socket size [11] with variable follow‐up times ranging from 3 to 60 days. Studies have reported conflicting outcomes with regard to healing of the tooth extraction socket in diabetic individuals [15, 16]. There is no consensus on the guidelines for the assessment of healing of extraction sockets, time for evaluation, and postoperative complications.

Given the high prevalence of periodontal diseases leading to tooth loss, establishing the relationship between diabetics and healing after tooth extraction is clinically relevant [17]. Even though good clinical practice guidelines recommend a coordinated approach between dentists and diabetologists or physicians to avoid any systemic complications, there has been a lack of consensus regarding the recommendations for extraction in diabetic patients [18]. This systematic review would help to summarize the existing studies related to healing of extraction sockets and postoperative complications to guide the clinicians in decision‐making and optimization of treatment plans and lay down the recommendations for future studies. Hence, the aim of this systematic review was to compare the healing of extraction sockets and postoperative complications between diabetics and controls. The objective was to compare the healing outcomes and postoperative complications, namely, dry socket, infection, and bony sequestra at maximal follow‐up.

## 2. Materials and Methods

This systematic review was conducted as per the “MOOSE guidelines” [19]. The protocol was registered in “PROSPERO” (CRD420251066405) [20].

### 2.1. Search Strategy

A systematic search was performed in “Scopus,” “PubMed,” “EMBASE,” and “Dentistry and Oral Sciences source” databases without any date restrictions on 31st August 2025. Search terms included a combination of diabetes OR diabetes mellitus OR T2DM OR T2D OR DM OR Diabetes OR insulin resistance OR non insulin dependent diabetes mellitus, tooth AND extraction OR teeth AND extraction OR tooth AND removal OR teeth AND removal OR exodontia OR Tooth Socket OR Wound Healing OR dental extraction OR healing socket OR Extraction socket. Filters used were adults, human, and English language (Table 1).

**Table 1:** Search strategy in different databases.

Scopus	ALL (tooth AND extraction OR teeth AND extraction OR tooth AND removal OR teeth AND removal OR exodontia OR tooth AND socket OR wound AND healing OR dental AND extraction OR healing AND socket) AND ALL (diabetes OR diabetes AND mellitus OR t2dm OR t2d OR dm OR diabetes OR insulin AND resistance OR non AND insulin AND dependent AND diabetes AND mellitus)
PubMed	(((diabetes[Title/Abstract] OR diabetic[Title/Abstract] OR diabetes mellitus[Title/Abstract] OR diabetic[Title/Abstract] OR T2DM[Title/Abstract] OR T2D[Title/Abstract] OR DM[Title/Abstract] OR Diabetes[Title/Abstract] OR insulin resistance[Title/Abstract] OR non insulin dependent diabetes mellitus[Title/Abstract]) OR ((diabetes mellitus[MeSH Terms]) OR (diabetes mellitus, insulin dependent[MeSH Terms]) OR (diabetes mellitus, non insulin dependent[MeSH Terms])) AND ((humans[Filter]) AND (english[Filter]) AND (alladult[Filter]))) AND ((tooth extraction[Title/Abstract] OR teeth extraction[Title/Abstract] OR dental extraction[Title/Abstract] OR tooth removal[Title/Abstract] OR teeth removal[Title/Abstract] OR exodontia[Title/Abstract] OR tooth socket[Title/Abstract] OR healing socket[Title/Abstract]) OR (extraction, tooth[MeSH Terms]) OR (tooth socket[MeSH Terms]) OR (exodontics[MeSH Terms]) AND ((humans[Filter]) AND (english[Filter]) AND (alladult[Filter]))) AND ((humans[Filter]) AND (english[Filter]) AND (alladult[Filter]))
Embase	(‘diabetes mellitus’/exp OR ‘non insulin dependent diabetes mellitus’/exp OR ‘insulin dependent diabetes mellitus’/exp) AND (‘tooth extraction’/exp OR ‘dental amputation’ OR ‘dental extraction’ OR ‘dental extractions’ OR ‘exodontia’ OR ‘exodontics’ OR ‘extraction, tooth’ OR ‘molar amputation’ OR ‘molar extraction’ OR ‘odontectomy’ OR ‘tooth amputation’ OR ‘tooth extraction’ OR ‘tooth removal’ OR ‘tooth resection’) AND ([adult]/lim OR [aged]/lim OR [very elderly]/lim) AND [humans]/lim AND [english]/lim
Dentistry and Oral Science	(diabetes OR diabetes mellitus OR T2DM OR diabetic OR T2D OR DM OR Diabetes OR insulin resistance OR non insulin dependent OR insulin dependent) AND (tooth AND extraction OR teeth AND extraction OR tooth AND removal OR teeth AND removal OR exodontia OR Tooth Socket OR dental extraction OR healing socket OR Extraction socket OR dental extraction sockets)

### 2.2. Inclusion and Exclusion Criteria

Studies were identified using the PICO framework. Population (P) comprised of patients undergoing tooth extraction; the intervention group (I) consisted of diabetic patients undergoing tooth extraction, compared against a control group (C) of nondiabetic patients undergoing the same procedure; and the outcome assessed (O) was socket healing parameters with or without complications. The review included interventional or observational studies published in English language that evaluated and compared the postextraction socket healing outcomes between diabetic and nondiabetic adults. Studies that had no comparator group, case reports, editorials or commentaries, conference abstracts, and non‐English publications were excluded.

### 2.3. Screening

Search results were added to “Rayyan”—a web‐based interface for screening and removal of duplicates [21]. Title and abstract screening was done by two clinicians independently—Kalyana C. Pentapati and Srikanth Gadicherla; any discrepancy was resolved by an arbitrator (Anupam Singh). The included studies were screened for full text by two trained review authors, Anupam Singh and Kalyana C. Pentapati, independently. Any discrepancy was resolved by an arbitrator (Mehul Saha).

### 2.4. Data Extraction

Data extraction was performed by two review authors, Anupam Singh and Kalyana C. Pentapati, independently. Information included was metadata of the studies, age and sex distribution, sample size, epithelization, granulation tissue, delayed wound healing, bony sequestra, dry socket, and infection for maximal follow‐up for both diabetes and control groups.

### 2.5. Risk of Bias

Quality assessment of all the included publications was done using the “Newcastle–Ottawa Quality Assessment Scale” by two authors—Kalyana C. Pentapati and Mehul Saha. It had three domains: selection (0–4), comparability (0–2), and exposure (0–3). The points (maximum of nine) were graded as high (≥7), moderate (5–6), and low quality (<5) [22].

### 2.6. Statistical Analysis

All the analyses were performed using “RevMan Ver.5.4.1 (Cochrane Collaboration, 2020).” Heterogeneity was assessed using the *Q* and *I* ^2^ statistics. Due to low heterogeneity, dichotomous variables (bone sequestra, delayed healing, and infection) were calculated using the Mantel–Haenszel method fixed‐effect model. Risk ratios and 95% confidence intervals were reported for each outcome. Publication bias and subgroup analysis were not performed owing to the less number of studies.

## 3. Results

**Figure 1:**
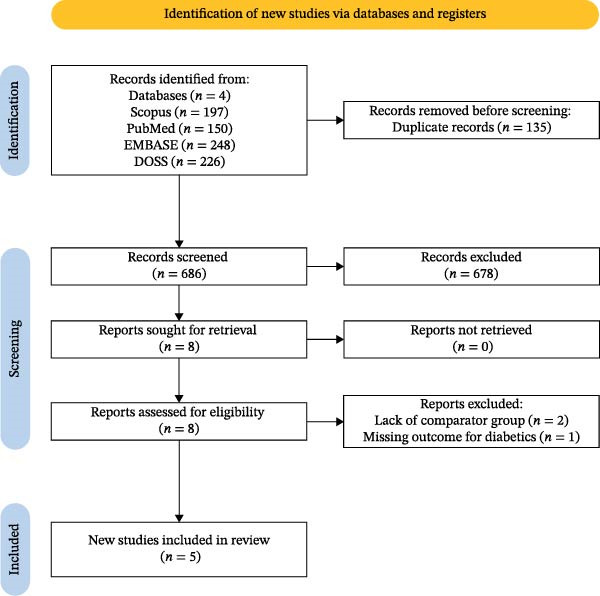
Detailing of the included studies for systematic review and meta‐analysis.

A comprehensive literature search was conducted across multiple electronic databases yielding a total of 821 results. After the removal of duplicates (*n* = 135), 686 unique publications were available for screening titles and abstracts. A total of eight publications were included for screening full text, of which only five publications [10–13, 15] were included for data extraction. Three publications were excluded, of which two publications did not have a comparator group [9, 14], and one publication did not report outcomes specific for diabetic patients [16] (Figure 1).

### 3.1. Characteristics of the Included Studies

A total of 500 diabetics and 364 controls were available from five included publications. The sample‐size–weighted mean age across included studies was 60.74 (range: 54.76–63.9) and 51.68 (46.8–59.6) years in the diabetic and control groups. The diabetic group included 288 males (57.6%), whereas the control group included 251 males (50.7%). The male‐to‐female ratio was 1.36:1 in the diabetic group and 1.03:1 in the control group (Table 2).

**Table 2:** Demographic characteristics of the included studies.

Author	Year	Group 1—sample size	Group 2—sample size	Group 1—age ± SD	Group 2—age ± SD	Group 1—male	Group 1—female	Group 2—male	Group 2—female
Huang et al.	2013	224	232	63.9^b^	46.8^b^	141	83	105	127
Fernandes et al.	2015	53	29	58 ± 11.14^a^	48 ± 11.16^a^	26	27	17	12
Power et al.	2019	56	49	58^b^	53^b^	26	30	26	23
Gadicherla et al.	2020	34	52	54.76 ± 13.58	54.04 ± 11.52	16	18	34	18
Krishnan et al.	2021	133	133	59.2 ± 9.6	59.6 ± 9.9	79	54	69	64

*Note:* Group 1—Diabetic study group. Group 2—Nondiabetic control group.

^a^Estimated from 95% CI.

^b^Standard deviation not reported.

### 3.2. Healing of Extraction Socket

Only one study reported the healing of extraction sockets in terms of socket dimensions at day 0 and day 7 [11]. Adjusted analysis reported that the mean socket size was larger among diabetics than the control group at the end of day 7.

### 3.3. Delayed Wound Healing

**Figure 2:**
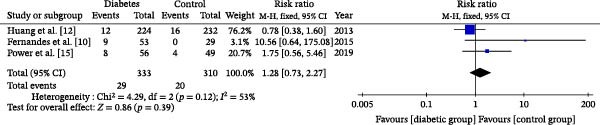
Meta‐analysis and forest plot of incidence of delayed wound healing.

Delayed wound healing was reported by three studies [10, 12, 15], of which two studies reported at the end of day 7 [12, 15] and one study reported at the end of day 21 [10]. Overall, there was no significant difference in the distribution of delayed wound healing among diabetics and controls (RR: 1.28; 95% CI: 0.73–2.27; *p* = 0.39; and *I* ^2^ = 53%) (Figure 2).

### 3.4. Dry Socket

Only one study reported the incidence of dry socket [13], in which the diabetic group (10/133) had a higher proportion than the control group (5/133).

### 3.5. Infection

**Figure 3:**
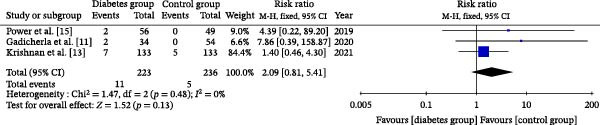
Meta‐analysis and forest plot of incidence of infection.

Three studies have reported the incidence of infection at the end of day 7 [11, 13, 15]. There was no difference in the incidence of infection between diabetes and control groups (RR: 2.09; 95% CI: 0.81–5.41; *p* = 0.13; and *I* ^2^ = 0%) (Figure 3).

### 3.6. Bony Sequestra

**Figure 4:**
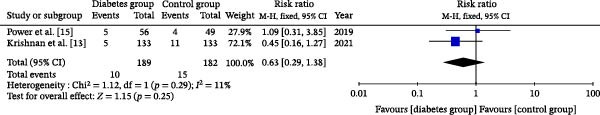
Meta‐analysis and forest plot of incidence of bony sequestra.

Two studies reported the incidence of bone sequestra [13, 15] at the end of day 7. There was no difference in the incidence of infection between diabetic and control groups (RR: 0.63; 95% CI: 0.29–1.38; *p* = 0.25; and *I* ^2^ = 11%) (Figure 4).

### 3.7. Risk of Bias Assessment

The risk of bias scores ranged from 3 to 7. None of the studies adjusted for the other potential confounders. All studies had an adequate selection of participants in both groups. One study matched case and control groups [15], and another study adjusted the preoperative socket dimensions while comparing the postoperative socket dimensions [11]. Overall, one study was high quality [11], one was low quality [13], and the other three were moderate quality [10, 12, 15] (Tables 3 and 4).

**Table 3:** Summary of risk of bias assessment of the included publications.

Study	Selection				Comparability	Exposure				Total
	Case definition	Representativeness	Selection of controls	Definition of controls		Ascertainment of exposure	Same methods for CC	Nonresponse rate	Exposure total	
Huang et al.	1	1	1	1	0	1	1	0	2	6
Fernandes et al.	1	1	1	1	0	0	1	0	1	5
Power et al.	1	1	1	1	2	0	0	0	0	6
Gadicherla et al.	1	1	1	1	1	1	1	1	3	8
Krishnan et al.	1	1	1	1	0	0	0	0	0	4

*Note:* ≥7 were considered high quality, 5–6 moderate quality, and <5 low quality.

**Table 4:** Summary of findings table.

Outcome	Number of studies	Total participants (diabetes/control)	Risk of bias	Inconsistency	Indirectness	Imprecision	Publication bias	Certainty of evidence (GRADE)
Dry socket	1	133/133	Serious^a^	No serious	No serious	Serious^b^	Unclear	⊕◯◯◯ Very low
Bony sequestra	2	189/182	Serious^a^	No serious	No serious	Serious^b^	Unclear	⊕◯◯◯ Very low
Delayed healing	3	333/310	Serious^a^	No serious	No serious	Serious^b^	Unclear	⊕◯◯◯ Very low
Infection	3	223/236	Serious^a^	No serious	No serious	Serious^b^	Unclear	⊕◯◯◯ Very low

^a^Downgraded for risk of bias due to methodological limitations (inadequate control for confounding).

^b^Downgraded due to small total sample sizes and/or low event counts, failure to meet optimal information size (OIS).

## 4. Discussion

This SRMA consolidated the available evidence on the healing of tooth extraction sockets and the incidence of complications among diabetics. Hyperglycemia can interfere with healing due to advanced glycation end‐products, vascular compromise, and reduced immune cell function. It has been proposed that due to the alteration in the microcirculation, the delivery of essential nutrients to the healing site gets affected. Subsequently, the whole wound healing cycle, i.e., the inflammatory phase, re‐epithelialization, and remodeling phase, is impacted, ultimately leading to delayed wound healing and increased chances of wound infection [23].

Due to these reasons, dental health care providers are vigilant in performing dental extractions and often defer these procedures. In addition, comparative studies report that uncontrolled diabetic patients undergoing oral surgical procedures are at increased risk of severe secondary space infections, with a greater number of spaces involved, more abnormal hematological findings, and a higher requirement for additional interventions like tracheostomy, compared with nondiabetic patients [24]. There have been numerous case reports implicating poorly controlled diabetes status as the contributing factor for the development of osteomyelitis or fungal infections like mucormycosis [25].

Healing after tooth extraction as an outcome has been poorly documented in the literature due to a lack of consensus on the assessment. In our review, we came across only a few studies that evaluated the incidence of complications like delayed wound healing, dry socket, bony sequestra, and infection. None of these outcomes showed a higher incidence among diabetes when compared to controls. Numerous factors like type of tooth, complexity of the procedure, glycemic levels, compliance of medication, use of antibiotic prophylaxis, comorbidities, and smoking can have substantial influence on the healing and complications [26–29]. The role of these factors was not completely investigated among the included studies.

The tooth extraction socket healing was assessed by a reduction in the socket size during the follow‐up period. The difference in socket healing between the diabetic and nondiabetic individuals can be attributed to the alteration in the angiogenesis process, along with the reduced collagen synthesis, which are essential components of extraction site healing [30].

Increased AGE deposition interferes with the cross‐linking of collagen, thereby affecting the stability of the extracellular matrix. Additionally, the microvascular changes have been shown to be detrimental to the wound healing capacity. A well‐functioning microcirculation maintains homeostasis. Not only does it provide the essential nutrients, but it also removes the waste products and modulates the inflammatory responses. Alteration in the basement membrane thickness of the capillaries affects their permeability. It impedes leucocyte migration and causes underperfusion, leading to tissue hypoxia [31, 32]. Even though these factors have been widely implicated as the reason for increased chances of wound infection, the pooled analysis of our review did not show any statistical significance between the two groups.

A dry socket generally presents with the features of dislodged clot, followed by debris accumulation inside the socket, leading the features of localized inflammation with or without concomitant infection. Local factors like traumatic extraction, decreased perfusion at the extraction site, and complexity of tooth extraction are some of the known risk factors. However, systemic conditions such as diabetes mellitus, although proposed as potential risk factors, have not been convincingly established as contributors to the development of dry socket [33]. The findings of this systematic review likewise do not demonstrate any association between diabetes and dry socket, primarily due to the lack of consistent reporting of dry socket across the included studies.

Although statistical heterogeneity was not a major problem, there was substantial variability in the assessment of the outcomes. Studies have not stratified the outcomes based on the glycemic status, and all the studies included those patients who had acceptable glycemic control. Importantly, because of this limiting factor, these findings should be interpreted as applying specifically to diabetic patients with controlled disease and should not be extrapolated to patients with poor glycemic control, who remain at risk of more severe complications, including life‐threatening deep neck space infections. There was heterogeneity in the assessment of the glycemic status. In view of the above factors, the observed result would have underestimated the role of diabetes in the healing process and complications after dental extractions. Another important consideration is the differentiation between the types of diabetes and their respective impact on socket healing. Healing outcomes and associated complications may vary between type 1 insulin‐dependent diabetes mellitus and type 2 diabetes mellitus. As a result, the observed result would have underestimated the role of diabetes in the healing process and complications after dental extractions.

There is broad consensus regarding the strong inter‐relationship between oral health and diabetes. The American Dental Association emphasizes on monitoring of the patient’s blood glucose prior to the procedure and stresses upon postprocedure nutrition of the patient to minimize any significant alteration in the patient’s blood glucose [34]. Similarly, the European Federation of Periodontology and the International Diabetes Federation have developed jointly the consensus guidelines. This aims at enhancing early diagnosis, prevention, and coordinated management of both diabetes and periodontitis [35]. However, in contrast to these established recommendations, there is a notable lack of consensus statements specifically addressing dental extractions and their associated complications in patients with diabetes. Hence, we propose evidence‐based clinical recommendations for tooth extraction in patients with diabetes mellitus, which can help clinicians in key decision‐making processes [30–34] (Table 5).

**Table 5:** Evidence‐based clinical recommendations for tooth extraction in patients with diabetes mellitus.

Stage	Recommendation
Glycemic assessment	Check HbA1c and/or random blood glucose preoperatively
	HbA1c < 7% considered optimal; proceed with standard protocol
	If poorly controlled (HbA1c ≥ 8% or fasting glucose > 180 mg/dL), defer elective extraction and optimize glycemic control in consultation with the patient’s diabetologist or physician [30, 31]
Medication and timing	Schedule appointments preferably in the morning
	Confirm that the patient has eaten normally and taken prescribed antidiabetic medications
	Chairside glucose monitoring (glucometer) is advisable
	Keep glucose source (e.g., juice and glucose gel) available to manage intraoperative hypoglycemia [32]
Antibiotic prophylaxis	Routine antibiotic prophylaxis is not recommended for well‐controlled or moderately controlled diabetic patients undergoing uncomplicated dentoalveolar extraction
	Consider perioperative antibiotic coverage if (a) diabetes is poorly controlled (fasting glucose > 250 mg/dL) or (b) acute odontogenic infection is present
	Decisions should be made individually; antibiotic stewardship principles apply [33]
Surgical technique	Use an atraumatic surgical technique to minimize tissue trauma and avoid delayed socket healing
	Maintain strict asepsis throughout the procedure
	Prescribe chlorhexidine 0.12%–0.2% oral rinses twice daily for 5–7 days postextraction to reduce infection risk and support wound healing [34]
Postoperative monitoring	Schedule an early clinical review at 5–7 days postextraction to assess socket healing
	Educate patients to report early warning signs: pain unresponsive to analgesics, increasing swelling, pus discharge, fever, or persistent bleeding
	If complications arise, perform socket irrigation and review/escalate antibiotic therapy as clinically indicated
	Note: Evidence from this review shows no statistically significant difference in delayed healing (RR: 1.28; *p* = 0.39) or infection (RR: 2.09; *p* = 0.13) between diabetic and nondiabetic patients
Patient education	Emphasize to patients that optimized glycemic control supports wound healing, thereby reducing the risk of infection
	Reinforce meticulous oral hygiene and regular use of prescribed antiseptic mouth rinses
	Encourage routine dental check‐ups and integrated care with the patient’s diabetologist
	Inform patients of the bidirectional relationship between diabetes and oral health; periodontal disease may adversely affect glycemic control [30]

*Note:* However, in contrast to these established recommendations, there is a notable lack of consensus statements specifically addressing dental extractions and their associated complications in patients with diabetes. Hence, we propose evidence‐based clinical recommendations for tooth extraction in patients with diabetes mellitus, which can help clinicians in key decision‐making processes (Table 5).

The strengths of the SRMA are that it included a comprehensive search across a wide range of databases available and it was conducted in accordance with the MOOSE guidelines. The independent screening and evaluation by two independent reviewers at every stage strengthened the methodological rigor of the review. However, the number of eligible studies was limited (*n* = 5), which produced a modest pooled sample size. Owing to the less number of studies, we could not evaluate the confounding effect of age on the overall outcomes. These studies also had considerable heterogeneity with regard to the study outcomes, assessment method, and follow‐up period. Further, the included studies were of only moderate methodological quality, and they had not adjusted for any of the confounding factors, which could have affected the study outcomes. These limitations reflect the need for more high‐quality, standardized, prospective studies, which will help to generate more definitive evidence.

## 5. Conclusion

In view of the above factors and the available evidence from the included studies, it can be concluded that, among patients with well‐controlled (acceptable) glycemic status, diabetes does not appear to be associated with poorer healing or an increased risk of complications after dental extraction. This conclusion should not be extrapolated to patients with poorly controlled diabetes, in whom oral surgical procedures may carry a risk of more severe outcomes, including life‐threatening deep neck space infections. Overall, there is a need for high‐quality studies that evaluate the healing, time taken for socket closure, and risk of complications. We also advocate for coordinated, multidisciplinary, and transnational efforts among relevant stakeholders to develop clear, evidence‐based guidelines to address this gap and clinical ambiguity. Within the limits of this review, it can be concluded that, in diabetic patients with acceptable glycemic control, dental extraction does not appear to impair healing or increase the risk of complications, albeit with very low certainty of evidence.

## Author Contributions

Conceptualization, methodology (data extraction), and writing – original draft: Anupam Singh and Kalyana C. Pentapati. Methodology (title and abstract screening): Srikanth Gadicherla, Kalyana C. Pentapati, and Anupam Singh. Methodology (full‐text screening): Kalyana C. Pentapati, Anupam Singh, and Mehul Saha. Methodology (risk of bias assessment): Kalyana C. Pentapati and Mehul Saha. Formal analysis: Kalyana C. Pentapati and Aditya Shetty. Review and editing: Premalatha K. Shetty, Aditya Shetty, and Srikanth Gadicherla. Project administration: Anupam Singh. Visualization: Srikanth Gadicherla, Premalatha K. Shetty, and Aditya Shetty.

## Funding

None of the authors received any specific funding for this study.

## Disclosure

All authors have read and approved the final version of the manuscript.

## Ethics Statement

This is not required since the study is a systematic review and meta‐analysis.

## Conflicts of Interest

The authors declare no conflicts of interest.

## General Statement

The corresponding author, Dr. Kalyana C. Pentapati, had full access to all of the data in this study and takes complete responsibility for the integrity of the data and the accuracy of the data analysis.

## Supporting Information

Additional supporting information can be found online in the Supporting Information section.

## Supporting information


**Supporting Information** MOOSE (Meta‐analyses Of Observational Studies in Epidemiology) Checklist.

## Data Availability

The authors confirm that the data supporting the findings of this study are available within the article and/or its supporting information. Additional data may also be obtained from the corresponding author upon request.
